# Weighting the United States *All of Us* Research Program data to known population estimates using raking

**DOI:** 10.1016/j.pmedr.2024.102795

**Published:** 2024-06-08

**Authors:** Vivian Hsing-Chun Wang, Jingwen Lei, Tingjia Shi, José A. Pagán

**Affiliations:** a Center for Population Health & Health Services Research, Department of Foundations of Medicine, NYU Grossman Long Island School of Medicine, Mineola, NY, USA; b Department of Biostatistics, School of Global Public Health, New York University, New York, NY, USA; c Department of Public Health Policy and Management, School of Global Public Health, New York University, New York, NY, USA

**Keywords:** Raking, Population health, Health equity, Health services research, *All of Us*

## Abstract

**Background:**

The *All of Us* Research Program aims to collect longitudinal health-related data from a million individuals in the United States. An inherent challenge of a non-probability sampling strategy through voluntary participation in *All of Us* is that findings may not be nationally representative for addressing health and health care at the population level. We generated survey weights for the *All of Us* data that can be used to address the challenge.

**Research design:**

We developed raked weights using demographic, health, and socioeconomic variables available in both the 2020 National Health Interview Survey (NHIS) and *All of Us*. We then compared the unweighted and weighted prevalence of a set of health-related variables (health behaviors, health conditions, and health insurance coverage) estimated from *All of Us* data with the weighted prevalence estimates obtained from NHIS data.

**Subjects:**

The sample included 100,391 *All of Us* participants 18 years of age and older with complete data collected between May 2017 and January 2022 across the United States.

**Results:**

Final variables in the raking procedure included age, sex, race/ethnicity, region of residence, annual household income, and home ownership. The mean percentage difference between known proportions obtained from the NHIS and *All of Us* was reduced by 18.89% for health-related variables after applying the raked weights.

**Conclusions:**

Raking improved the comparability of prevalence estimates obtained from *All of Us* to known national prevalence estimates. Refining the process of variable selection for raking may further improve the comparability between *All of Us* and nationally representative data.

## 1 Introduction

The *All of Us* Research Program is a key component of the Precision Medicine Initiative that was launched under the direction of the White House in the United States (US) in 2015 (The Precision Medicine Initiative, 2023). The initiative leverages advances in science and technology to develop new health care models that take into account variability between individuals in genetics, the environment, socioeconomic conditions, and lifestyles (Denny et al., 2019). The goal of *All of Us* is to develop a longitudinal database that integrates information from self-reported surveys, electronic health records (EHRs), genomic data, physical measurements, and other health-related data from at least one million individuals (Denny et al., 2019).

A core principle of *All of Us* is that it prioritizes reaching populations that are historically underrepresented in biomedical research (UBR) (Mapes et al., 2020). UBR populations include members of racial/ethnic minority groups as well groups defined by factors such as age, sex, gender, socioeconomic status, access to health care services, rurality, and/or disability (Mapes et al., 2020). The *All of Us* Research Program has developed strategies for recruitment that include engagement efforts with community partners to conduct outreach with UBR groups (Mapes et al., 2020, Lyles et al., 2018, Mercer et al., 2018). Despite the intentional efforts to recruit UBR populations, the COVID-19 pandemic and other recruitment challenges have made it difficult for the *All of Us* Research Program to reach the projected enrollment level of participants and especially UBR populations. Recruitment and engagement with participants during COVID-19 was limited as a result of lack of access to in-person activities (Hedden et al., 2023). *All of Us* modified strategies for recruitment and the collection of biospecimen data, physical measurements, and surveys, so that some activities could take place remotely while also following safety guidelines from the Centers for Disease Control and Prevention (Hedden et al., 2023). Consent to participate in *All of Us* fell by 53 % in Black/African American adults and increased by 29 % for White adults before and after March 2020 when the COVID-19 pandemic hit the US (Heddedn et al., 2022). Socioeconomically disadvantaged populations were also less likely to participate in the study than the rest of the population (Hedden et al., 2023).

The objective of this study is to generate survey weights that can facilitate the use of *All of Us* data for population-based research. Developing strategies to calibrate *All of Us* data is crucial to make sure estimates obtained from the database are consistent with known population estimates. Understanding the patterns of health and diseases at the population level can help inform policies and programs needed to expand the utility of precision medicine at scale. Raking is particularly useful for estimating survey weights because access to *All of Us* data is highly protected through the use of a cloud-based platform. This data management approach protects data security and participant privacy but does not allow the use of data management and analytical/statistical software outside the *All of Us* cloud-based platform. Raking provides a convenient way to integrate population-level data (i.e., known population estimates/proportions for different categorical variables) with protected individual-level data to estimate survey weights.

## 2 Materials and methods

### 2.1 Data source

*All of Us* began its data collection in May 2017 and the program has recruited over 630,000 individuals as of April 30, 2023. Persons 18 years of age and older living in the US or a territory of the US with the “legal authority and decisional capacity to consent” in either English or Spanish were eligible to participate in the program (All of Us Research Program Operational Protocol, 2021). Participants included in the database had provided consent to participate in the study, authorized the sharing of EHR data, completed baseline surveys, and provided physical measurements and at least one biospecimen for biobank storage. Key characteristics of the *All of Us* data are summarized in Table 1.

**Table 1:** Features of the *All of Us* Research Program.

**Data Description**	
*Data type*	Longitudinal (data collection began in May 2017 and it is ongoing)
*Data sources*	Self-reported surveys, electronic health records (EHRs), physical measurement, biospecimen, and data from wearable devices
*Sample size*	As of May 2023, more than 630,000 participants enrolled in the program; the goal is to enroll over 1 million participants within 5 years
*Inclusion criteria*	Adults 18 years of age and older with the capacity to consent who currently live in the US or a US territory; children are expected to be eligible for participation in 2023
*Exclusion criteria*	Individuals incarcerated at the time of enrollment
*Geographic coverage*	All 50 states, 5 US territories, 3 freely associated states, and the District of Columbia
*Data access*	Data accessed through the Researcher Workbench, a cloud-based computing platform (Jupyter Notebook) that provides users access to R, Python, SAS, SQL, and other tools for data analytics
*Data privacy*	De-identified data provided with generalization (e.g., 3-digit ZIP codes) or suppression (e.g., suppressing racial/ethnic subcategories) to protect the privacy of participants
*Survey topics*	Demographic and socioeconomic characteristics, lifestyle, self-reported health status, personal medical history, family health history, health care access and utilization, and social determinants of health

**Participant Engagement**	
*Participant engagement*	Participant representatives engage as true partners of the Research Program and serve on steering, research, and other governing committees
*Recruitment approach*	Remote (online) or in-person enrollment through health care provider organizations and community partners
*Participant diversity*	Emphasis on populations underrepresented in biomedical research (UBR): non-White, 65 years of age and older, intersex, identified with neither man nor woman, low income, without a high school diploma, no health insurance coverage, living in rural area, with a physical or cognitive disability, or pregnant

**Benefits and Limitations of using *All of Us***	
*Benefits*	Large sample size to study different populations; integrated longitudinal health data from multiple sources; geographic identifiers (3-digit ZIP codes)
*Limitations*	Participant recruitment is not population-based; survey data were not collected on a set schedule; responses to some survey questions are not updated frequently (e.g., home ownership, annual household income, employment status)

### 2.2 Benchmark data used in the raking procedure

We used the 2020 NHIS to identify benchmark variables as the population parameters to be included in the raking procedure. NHIS is an annual, in-person survey collected from a sample representative of US households. Persons institutionalized in long-term care or correctional facilities or on active military duty were excluded from participation. NHIS uses a multistage probability sampling design for sample selection and collects information from about 100,000 individuals every year.

### 2.3 Analytic sample

Participation in the different survey modules is voluntary and takes place at the pace of the respondents. As such, we selected respondents with complete data in all variables of interest, which resulted in an analytic sample of 100,391 *All of Us* adult participants residing in the US. Data included in this study were released on June 23, 2022 (this data release includes participants that provided data up to January 1, 2022) (Master et al., 2022).

### 2.4 Measures

*Variables for raking.* Variables available in both *All of Us* and the NHIS were considered for the raking procedure. The variables included were age group (18–24, 25–34, 35–44, 45–54, 55–64, 65 years of age and older), biological sex at birth (female, male), race/ethnicity (non-Hispanic White, non-Hispanic Black, non-Hispanic Asian, Hispanic, Others), Census region of residence (Northeast, Midwest, South, West), annual household income (less than $25,000, $25,000 to less than $35,000, $35,000 to less than $50,000, $50,000 or more), educational attainment (did not graduate high school, graduated high school, some college, college graduate), home ownership status (own, rent, other arrangement), sexual orientation (gay/lesbian/bisexual/something else, straight), self-reported health status (excellent, very good, good, fair, poor), usual place of care (doctor’s office, clinic or health center, urgent care or minute clinic, hospital emergency room, some other place or don’t go to one place most often, don’t have a usual place for care), and interval since last doctor’s visit (less than 1 year, 1 year to less than 2 years, more than 2 years).

*Outcome variables.* We selected key lifestyle risk factors, associated chronic health conditions, and health insurance coverage to compare the difference between the *All of Us* estimates applying raked weights and the national prevalence values estimated from the NHIS. Lifestyle risk factors included alcohol use (yes/no) and being ever a smoker (yes/no). Associated chronic health conditions were identified through separate survey questions asking respondents whether they had hypertension, coronary artery disease, and diabetes.

### 2.5 Statistical analyses

We applied iterative proportional fitting (IPF, known also as raking) to estimate survey weights for the cohort of US adults included in our analytic sample. We followed the general approach used to compute sampling weights for the survey datasets of the American National Election Studies (ANES) (DeBell and Krosnick, 2009, Pasek et al., 2014). Participation in the *All of Us* Research Program is voluntary and, as such, weighting the data following the generally recommended approach for the ANES survey datasets may prove useful to adjust the data to known population values.

We first identified categorical variables comparable between the study proportions to known population proportions in NHIS (Deville et al., 1993). These variables were described in the ***Measures*** section above. In raking, the study proportions were compared to the known proportions one variable at a time, and they were adjusted by multiplying existing sampling weights by the ratio of the known proportion and the study proportion. The process was applied sequentially to each of the selected variables, adjusting the existing weight calculated in the previous step with a new weight adjusted for the new variable being considered in the raking process. The process was repeated until the weighted proportions for all the variables considered were as close as possible to the known proportions for all the variables (this was accomplished using a prespecified variable discrepancy tolerance level) (Deville and Sarndal, 1992). The final selection of variables to be included in constructing weights via raking were based on eliminating bias (i.e., the difference between the *All of Us* study prevalence estimates and the benchmark values obtained from the NHIS). In following the guidance developed by the ANES committee, we excluded variables with less than a five percentage point discrepancy (Pasek et al., 2014). We used the anesrake R package (version 0.80; available at the Comprehensive R Archive Network) to implement the raking procedure.

*All of Us* data were accessed through the Researcher Workbench, a cloud-based platform that supports data analysis and collaboration in a Jupyter Notebook environment. All data management and statistical analyses were conducted using Structured Query Language (SQL) and the R (version 4.2.1) software environment for statistical computing. *All of Us* participants provided consent to participating in the study, the protocol of which was approved and is actively monitored by the *All of Us* Institutional Review Board (IRB). All data in *All of Us* was de-identified; therefore, the requirement for institutional IRB review was waived for this study.

## 3 Results

Table 2a presents a comparison of population proportions of NHIS and study proportions of *All of Us*, both unweighted and weighted with raked weights. The variables used in the raking procedure for this study included age, sex, race/ethnicity, region of residence, annual household income, and home ownership. The mean percentage difference between the NHIS and unweighted *All of Us* estimates was 6.69 %. When compared to NHIS estimates, the unweighted age distribution of *All of Us* had a lower proportion of younger adults and a higher proportion of older adults; a higher proportion of female, gay, lesbian, bisexual, and other, non-Hispanic White adults, college graduates, participants with other living arrangement, residence in the Northeast, Midwest, and West regions, self-reported health status as very good, good, or fair, individuals having doctor’s office, clinic or health center as a usual source of care, and individuals with a visit to the doctor in less than a year.

**Table 2a:** Demographic Profile of the 2020 National Health Interview Survey and estimates for adults 18 + from the May 2017–January 2022 *All of Us* Research Program using unweighted data and data with raked weights (N = 100,391).

**Characteristic**	***All of Us* sample size**	**2020 NHIS, % (N = 35,399)**	**Unweighted prevalence estimate (%), 95 %CI**		**Absolute percentage difference**	**Weighted** **prevalence estimate (%), 95 %CI**		**Absolute percentage difference**
Age group*								
18–24	4,224	11.60	4.21	4.09–4.33	7.39	11.59	11.23–11.96	0.01
25–34	14,239	17.70	14.18	13.97–14.40	3.52	17.68	17.34–18.03	0.02
35–44	13,968	16.40	13.91	13.70–14.13	2.49	16.38	16.06–16.71	0.02
45–54	14,964	15.80	14.91	14.69–15.13	0.89	15.78	15.48–16.09	0.02
55–64	21,097	16.90	21.01	20.76–21.27	4.11	16.88	16.60–17.17	0.02
65+	31,899	21.70	31.77	31.49–32.06	10.07	21.68	21.38–21.98	0.02

Sex*								
Male	34,520	48.40	34.39	34.09–34.68	14.01	48.40	47.97–48.83	0.00
Female	65,871	51.60	65.61	65.32–65.91	14.01	51.60	51.17–52.03	0.00

**Sexual orientation**								
Straight	89,766	96.10	89.42	89.22–89.61	6.68	87.34	87.02–87.64	8.76
Bisexual/Gay/Lesbian/Other	10,625	3.90	10.58	10.39–10.78	6.68	12.66	12.36–12.98	8.76

Race/ethnicity*								
Non-Hispanic White	78,233	63.60	77.93	77.67–78.18	14.33	63.60	63.15–64.05	0.00
Non-Hispanic Black	7,674	11.30	7.64	7.48–7.81	3.66	11.30	11.00–11.61	0.00
Non-Hispanic Asian	3,393	5.90	3.38	3.27–3.49	2.52	5.90	5.67–6.14	0.00
Hispanic	8,689	16.50	8.66	8.48–8.83	7.84	16.50	16.12–16.89	0.00
Other	2,402	2.70	2.39	2.30–2.49	0.31	2.70	2.56–2.85	0.00

Educational attainment*								
Did not graduate high school	1,784	11.60	1.78	1.70–1.86	9.82	1.96	1.84–2.09	9.64
High school graduate	8,214	28.10	8.18	8.01–8.35	19.92	8.77	8.52–9.04	19.33
Some college	23,440	30.60	23.35	23.09–23.61	7.25	23.66	23.29–24.04	6.94
College graduate	66,953	29.80	66.69	66.40–66.98	36.89	65.60	65.18–66.02	35.80

Annual household income from all sources*								
Less than $25,000	14,734	13.80	14.68	14.46–14.90	0.88	13.80	13.50–14.11	0.00
$25,000 to less than $35,000	7,310	8.80	7.28	7.12–7.44	1.52	8.80	8.54–9.07	0.00
$35,000 to less than $50,000	10,091	12.70	10.05	9.87–10.24	2.65	12.70	12.39–13.01	0.00
$50,000 or more	68,256	64.70	67.99	67.70–68.28	3.29	64.70	64.27–65.12	0.00

Do you own or rent the place where you live?*								
Own	65,605	68.20	65.35	65.05–65.64	2.85	68.20	67.79–68.61	0.00
Rent	28,611	29.60	28.50	28.22–28.78	1.10	29.60	29.20–30.00	0.00
Other living arrangement	6,175	2.20	6.15	6.00–6.30	3.95	2.20	2.11–2.30	0.00

Region of residence*								
Northeast	29,639	17.40	29.52	29.24–29.81	12.12	17.40	17.14–17.66	0.00
Midwest	27,970	21.10	27.86	27.58–28.14	6.76	21.10	20.80–21.40	0.00
South	18,139	37.70	18.07	17.83–18.31	19.63	37.70	37.25–38.16	0.00
West	24,643	23.80	24.55	24.28–24.81	0.75	23.80	23.45–24.15	0.00

**Self-reported health status**								
Excellent	13,809	24.40	13.76	13.54–13.97	10.64	14.22	13.92–14.53	10.18
Very good	38,798	34.70	38.65	38.35–38.95	3.95	38.65	38.23–39.07	3.95
Good	31,755	27.50	31.63	31.34–31.92	4.13	32.07	31.67–32.47	4.57
Fair	13,250	10.60	13.20	12.99–13.41	2.60	12.54	12.26–12.82	1.94
Poor	2,779	2.90	2.77	2.67–2.87	0.13	2.52	2.40–2.66	0.38

**Usual place for care**								
Doctor’s office, clinic or health center	87,330	79.40	86.99	86.78–87.20	7.59	83.86	83.51–84.20	4.46
Urgent care or minute clinic	5,566	6.90	5.54	5.40–5.69	1.36	6.68	6.45–6.92	0.22
Hospital emergency room	1,041	1.40	1.04	0.98–1.10	0.36	1.17	1.07–1.27	0.23
Some other place	2,247	2.60	2.24	2.15–2.33	0.36	2.43	2.29–2.57	0.17
Don't have a usual place for care	4,207	9.70	4.19	4.07–4.32	5.51	5.87	5.64–6.10	3.83

**Interval since last doctor's visit**								
Less than 1 year	95,174	83.30	94.80	94.66–94.94	11.50	92.78	92.52–93.02	9.48
1 year to less than 2 years	3,655	9.20	3.64	3.53–3.76	5.56	4.81	4.61–5.01	4.39
More than 2 years	1,562	7.50	1.56	1.48–1.63	5.94	2.42	2.27–2.58	5.08

*Variables used in raking: age, sex, race/ethnicity, region of residence, annual household income from all sources, and home ownership.

Table 2b reports the prevalence of health and health behavior outcomes for *All of Us* adults estimated using unweighted data and data with raked weights. The mean percentage difference between NHIS and *All of Us* after applying raked weights was 3.21 %. Applying the raked weights to socioeconomic and demographic characteristics reduced the mean percentage difference between known proportions obtained from the NHIS and *All of Us* by 51.94 % (i.e., from 6.69 % to 3.21 %) among demographic variables and by 18.89 % (i.e., from 6.25 % to 5.07 %) among health and health behavior outcome variables (hypertension, coronary artery disease, diabetes mellitus, alcohol use, ever smoker, and health insurance coverage).

**Table 2b:** Prevalence of health and health behavior outcomes for adults 18 + from the May 2017–January 2022 *All of Us* Research Program estimated using unweighted data and data with raked weights (N = 100,391)*.

**Characteristic**	***All of Us* sample size**	**2020 NHIS, % (N = 35,399)**	**Unweighted prevalence estimate (%), 95 %CI**		**Absolute percentage difference**	**Weighted** **prevalence estimate (%), 95 %CI**		**Absolute percentage difference**
**Hypertension**	32,287	31.20	32.16	31.87–32.45	0.96	29.20	28.82–29.57	2.00
**Coronary artery disease**	4,585	4.70	4.57	4.44–4.70	0.13	4.03	3.88–4.18	0.67
**Diabetes mellitus**	11,712	9.30	11.67	11.47–11.87	2.37	10.99	10.74–11.25	1.69
**Alcohol use**	96,095	86.60	95.72	95.59–95.84	9.12	94.23	94.00–94.45	7.63
**Ever smoker**	35,282	16.10	35.14	34.85–35.44	19.04	30.13	29.76–30.51	14.03
**Covered by health insurance**	95,119	88.90	94.75	94.61–94.88	5.85	93.27	93.03–93.50	4.37
Mean percentage difference between NHIS and unweighted *All of Us* (%)								6.25
Mean percentage difference between NHIS and raked *All of Us* (%)								5.07
Absolute change in mean percentage difference after raking (%)								1.18
Relative change in mean percentage difference after raking (%)								18.89

*Variables used in raking: age, sex, race/ethnicity, region of residence, annual household income from all sources, and home ownership.

## 4 Discussion

Our findings suggest that applying raked weights can better align prevalence estimates obtained from *All of Us* data with prevalence estimates obtained from the NHIS for health-related behaviors, conditions, and health insurance coverage. In this study we were able to reduce the mean percentage difference between known proportions obtained from the NHIS and *All of Us* by one fifth of the mean percentage difference. The reduction in bias was obtained by calculating raked weights based on age, sex, race/ethnicity, region of residence, annual household income, and home ownership variables.

The Pew Research Center conducted a comprehensive weighting study with 30,000 online opt-in panel interviews with three commercial panel vendors (the vendors were not named in the study) (Mercer et al., 2018). The purpose of the study was to compare weighting using raking, propensity score weighting, and matching. Variables considered in the weighting procedure included demographic variables (age, sex, race/ethnicity, education, and region of residence) and other variables related to political attitudes (voter registration, political party, and religion). The study evaluated different sample size scenarios (up to 8,000 interviews) and found an 8.4 percentage point difference between 24 benchmark variables from the American Community Survey (and other gold standard surveys such as the Current Population Survey and the General Social Survey (GSS)) and the unweighted variables from the opt-in panel surveys. Bias was reduced by about 30 % with the three weighting methods, and the differences between them were very small. The study concluded that accuracy can be improved by carefully choosing the right variables for weighting and adding political variables (i.e., variables related to the main topic of interest in the survey work), and that raking performs as well as propensity score weighting and matching (Mercer et al., 2018).

Other studies have come to similar conclusions. An online opt-in survey (n = 1,017) consisting of 49 multiple-choice attitudinal questions from the GSS and random-digit dialing surveys from the Pew Research Center found that the median absolute difference between the opt-in (non-probability) and probability surveys were 9.1 percentage points; statistical adjustment using raking reduced this to 7 percentage points (Goel et al., 2017). A study comparing raking and poststratification adjustments for the 2013 South Australian Monitoring and Surveillance System (n = 7,193) found that raked weights (to adjust the data to the 2011 Census) substantially improved the accuracy of prevalence estimates for health conditions and behavioral risk factors (Dal Grande et al., 2015). The study concluded that raked weights calculated using demographic variables as well as dwelling status (home ownership) and other variables reduce nonresponse bias and incorporate “lower socioeconomic groups and those who are routinely not participating in population surveys into the weighting formula” (Dal Grande et al., 2015).

Applying raked weights can improve the accuracy of estimates obtained from *All of Us* but it can also lead to more variation around mean prevalence estimates. Other important considerations include minimizing the difference between the data collection date of *All of Us* variables and the reference date of the benchmark value variable, addressing missing data, making sure that there are enough *All of Us* participants in each category of each variable to obtain meaningful estimates, and considering whether there is a need to develop raked weights that vary over time given that each year more participants join the *All of Us* Research Program and the study has been collecting data on participants beginning in 2017. Still, the approach conducted here is useful given the non-probability sampling approach used in *All of Us*, the difficulties faced by the *All of Us* Research Program to collect data during the COVID-19 pandemic, and the data protection and access limitations in place that do not allow the use of data management and analytical/statistical software outside the *All of Us* Researcher Workbench.

## Funding sources

This research study did not receive any specific grant from funding agencies in the public, commercial, or not-for-profit sectors.

## CRediT authorship contribution statement

**Vivian Hsing-Chun Wang:** Writing – review & editing, Writing – original draft, Methodology, Formal analysis, Conceptualization. **Jingwen Lei:** Writing – review & editing, Formal analysis, Data curation. **Tingjia Shi:** Writing – review & editing, Formal analysis, Data curation. **José A. Pagán:** Writing – review & editing, Writing – original draft, Methodology, Formal analysis, Conceptualization.

## Declaration of competing interest

The authors declare that they have no known competing financial interests or personal relationships that could have appeared to influence the work reported in this paper.

## Data Availability

This study used data from the *All of Us* Research Program’s Controlled Tier Dataset v7, available to authorized users on the Researcher Workbench.
